# A new *Drosophila melanogaster* research resource: CRISPR-induced mutations for clonal analysis of fourth chromosome genes

**DOI:** 10.1093/g3journal/jkaf006

**Published:** 2025-01-13

**Authors:** Bonnie M Weasner, Brandon P Weasner, Kevin R Cook, Michael J Stinchfield, Shu Kondo, Kuniaki Saito, Justin P Kumar, Stuart J Newfeld

**Affiliations:** Department of Biology, Indiana University, 1001 E. Third St., Bloomington, IN 47405, USA; Department of Biology, Indiana University, 1001 E. Third St., Bloomington, IN 47405, USA; Bloomington Drosophila Stock Center, Department of Biology, Indiana University, 1001 E. Third St., Bloomington, IN 47405, USA; School of Life Sciences, Arizona State University, Tempe, AZ 85287, USA; Invertebrate Genetics Laboratory, National Institute of Genetics, Research Organization of Information and Systems, 1111 Yata, Mishima, Shizuoka 411-8540, Japan; Department of Biological Science and Technology, Tokyo University of Science, 6-3-1 Niijuku, Katsushika-ku, Tokyo 125-8585, Japan; Invertebrate Genetics Laboratory, National Institute of Genetics, Research Organization of Information and Systems, 1111 Yata, Mishima, Shizuoka 411-8540, Japan; Department of Biology, Indiana University, 1001 E. Third St., Bloomington, IN 47405, USA; School of Life Sciences, Arizona State University, Tempe, AZ 85287, USA

**Keywords:** *Drosophila melanogaster*, CRISPR, fourth chromosome, mutation, Fourth Chromosome Resource Project

## Abstract

As part of an ongoing effort to generate comprehensive resources for the experimental analysis of fourth chromosome genes in *Drosophila melanogaster*, the Fourth Chromosome Resource Project has used CRISPR mutagenesis with single guide RNAs to isolate mutations in 62 of the 80 fourth chromosome, protein-coding genes. These mutations were induced on a fourth chromosome bearing a basal FRT insertion to facilitate experimental approaches involving FLP recombinase-induced mitotic recombination. To permit straightforward comparisons among mutant stocks, most of the mutations were generated on isogenic fourth chromosomes, which were then crossed into a common genetic background. Of the 119 mutations, 84 are frameshift mutations likely to be null alleles, 29 are small, in-frame deletions, and 6 have yet to be characterized molecularly. The mutations were tested for recessive lethal, female-sterile, and visible phenotypes. Stable stocks for most of the mutations have been submitted to repositories in the United States and Japan for public distribution.

## Introduction

The specific roles a gene plays in the normal functioning of a cell are often inferred from the phenotypes seen when its activity is reduced or eliminated, and loss-of-function mutations are important for such experimental approaches. While the functional significance of a gene can sometimes be determined from phenotypes seen in individuals composed entirely of homozygous mutant cells, it is often more informative to examine the phenotypes of homozygous mutant cells embedded in an otherwise heterozygous individual. Indeed, experimental approaches involving mitotic recombination to generate small clones of mutant cells are particularly important when homozygosity results in zygotic lethality.

Most current clonal analysis experiments in Drosophila involve the use of the GAL4–UAS–GAL80 cell-specific expression system to control FLP recombinase-induced mitotic recombination between FRT sequences positioned at the bases of chromosome arms. These methods permit clone formation to be targeted spatially and temporally, and they allow investigators to determine the contributions of individual genes to the development, homeostasis, and adaptation of specific tissues (Weasner *et al.* 2017). These methods have become especially important in exploring cell-to-cell communication. For example, they are used extensively in studying competition among cells (Kiparaki and Baker 2023).

Here, we describe the efforts of the Fourth Chromosome Resource Project to develop Drosophila stocks for the clonal analysis of loss-of-function phenotypes for the majority of fourth chromosome, protein-coding genes. This work was prompted by the paucity of research resources related to the fourth chromosome and by 2 experimental developments. First, Goldsmith *et al*. (2022) used CRISPR site-specific genome-editing technology to insert an FRT sequence at the base of the long arm of the fourth chromosome (the short arm carries no genes), which they then used to generate mitotic clones. Second, a set of stocks bearing guide RNA-expressing transgenes for most protein-coding genes on the fourth chromosome was generated at the National Institute of Genetics in Japan. We recognized that the easiest way to place mutations distal to this basal FRT was to use these guide RNAs to induce mutations directly on the FRT-bearing chromosome. To add experimental value to the stock collection, we have taken care to generate the mutations on fourth chromosomes derived from a single progenitor chromosome and to place the mutagenized chromosomes into a common genetic background. The new mutation-bearing stocks are now being distributed by stock centers in both Japan and the United States.

## Materials and methods

An extensive description of our experimental methods is provided in Supplementary File 1. It provides details of the screens isolating CRISPR-induced mutations on a single-origin fourth chromosome carrying the FRT transgene *TI{TI}FRT101F* (Goldsmith *et al.* 2022); the crosses placing the mutagenized chromosomes into a common, isogenic background; the construction of stocks necessary for these crosses; the molecular characterization of the mutations; and the preliminary characterization of associated phenotypes. Mutations in 4 genes were generated by alternative crosses, which are also outlined. Supplementary Table 1 gives a list of fourth chromosome genes. Supplementary Table 2 provides details of the guide RNA transgenes used in the screens. Supplementary Table 3 gives primers utilized to identify and characterize new mutations. Supplementary Table 4 is the Reagent Table listing the progenitor stocks used to develop the strains employed in the screens, the stocks used to characterize mutant phenotypes, and the sources of biological and chemical reagents.

All fly cultures were reared under standard conditions. All genomic coordinates cited in this report refer to *D. melanogaster* genome release 6. Gene models, annotations, and nomenclature reflect FlyBase release 2024_4. Information about the new mutations in publicly distributed stocks has been archived in FlyBase (Öztürk-Çolak *et al.* 2024). Information about the stocks may be obtained via the websites of the Bloomington Drosophila Stock Center at Indiana University (https://bdsc.indiana.edu/) and the Department of Drosophila Genomics and Genetic Resources at the Kyoto Institute of Technology (https://www.dgrc.kit.ac.jp/). A preliminary report of this work was presented by Stinchfield *et al.* (2024).

## Results and discussion

As mentioned, our effort to generate CRISPR-induced mutations in fourth chromosome genes on an FRT-bearing chromosome depended on the availability of a set of guide RNA-expressing stocks from the Japan National Institute of Genetics. These stocks were generated as part of a large-scale project to construct guide RNA-expressing stocks for all *D. melanogaster* protein-coding genes, which will be described elsewhere. The transgenes in these stocks were constructed by the methods of Kondo and Ueda (2013), and they ubiquitously express guide RNAs with 20-base targeting sequences under the control of *snRNA:U6:96Ab* regulatory sequences. Stocks targeting 77 of the 80 protein-coding genes on the fourth chromosome were available for our efforts (those we used are listed in Supplementary Table 2). In addition, we used 1 guide RNA stock originating in the work of Zirin *et al*. (2020).

**Fig. 1.**
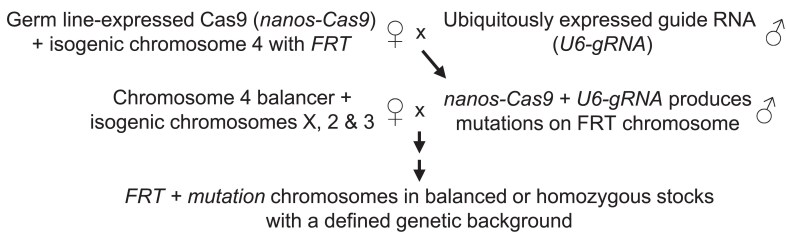
Conceptual outline of the CRISPR screens producing new fourth chromosome mutations. In males, a transgene expressing Cas9 in the germ line (*nanos-Cas9*) was combined with a transgene ubiquitously expressing a guide RNA targeting a specific fourth chromosome gene (*U6-gRNA*) and a transgene carrying an FRT sequence inserted near the base of an isogenized fourth chromosome. Many of the gametes produced by these males carried mutations in the targeted gene caused by Cas9-induced double-stranded DNA breaks. A series of crosses involving stocks with balancer chromosomes and isogenic X, second, and third chromosomes produced balanced or homozygous stocks with a defined genetic background. Supplementary File 1 provides a detailed description of the screens and the development of the stocks used.

As outlined in Fig. 1 and shown in detail in Supplementary File 1, the guide RNA-expressing transgenes were combined in males with a transgene expressing the Cas9 nuclease in the germ line and a fourth chromosome carrying the FRT transgene *TI{TI}FRT101F*. All FRT*-*bearing chromosomes used in screens were derived from a single chromosome to minimize sequence variation across all screens. Putative mutation-bearing fourth chromosomes were isolated in balanced or homozygous stocks and tested for alterations at the guide RNA target sites by PCR amplification followed by Sanger sequencing. We generally found it necessary to sequence fewer than 10 mutagenized chromosomes per gene to identify 1 or more independent mutations. Table 1 summarizes the results of the 69 screens undertaken using this method and Supplementary Table 5 describes the mutations chosen for phenotypic characterization. In several instances, we recovered independent mutations with identical molecular lesions—reflecting the predictability of CRISPR mutagenesis and DNA repair (Table 1).

**Table 1.** Recovery of fourth chromosome mutations in screens.

Gene	Mutagenized chromosomes recovered*^a^*	Frameshift mutations	In-frame deletions	No mutation
**Primary screening approach*^b,c^***				
*4E-T*	3	2	0	0
*Abcd1*	7	3	1	0
*Actbeta*	2	1	1	0
*Ank*	5	1	0	0
*anne*	3	3	0	0
*apolpp*	4	3 Identical	1	0
*Arf4*	4	1	0	0
*Arl4*	2	0	1	0
*ATPsynbeta*	5	1	0	1
*Cadps*	8*^d^*	1	0	3
*Cals*	4	0	1	2
*CaMKI*	8	2	0	0
*CG11360*	5	1	0	0
*CG1674*	4	2	1	0
*CG1909*	2	1	1	0
*CG31998*	3	2	0	0
*CG31999*	4	3 (2 identical)	1	0
*CG32017*	8	2	1	4
*CG33521*	3	3 Identical	0	0
*CG33978*	1	1	0	0
*ci*	4	1	2	0
*Crk*	11	4 Identical	1	6
*dpr7*	3	0	2 Identical	0
*Dyrk3*	3*^d^*	0	0	0
*eIF4G1*	6	2	1	3
*Ekar*	2	0	2	0
*Eph*	5	4 (2 identical)	0	0
*Ephrin*	1	1	0	0
*ey*	5	5 (3 identical)	0	0
*fd102C*	3	1	0	0
*Gyf*	7	2	0	2
*Hcf*	5	3 Identical	2 Identical	0
*Kif3C*	2	0	1	1
*lgs*	3	1	1	1
*mav*	4	2	0	2
*MED26*	3	0	2 Identical	1
*mGluR*	4	4	0	0
*Mitf*	3*^d^*	0	0	0
*myo*	3	1	2 Identical	0
*ND-49*	3	0	3 Identical	0
*NfI*	5	5 (2 identical)	0	0
*onecut*	4	3	0	1
*pan*	2	2	0	0
*pho*	5	1	0	2
*PMCA*	4	1	0	1
*Pur-alpha*	5	1	0	4
*Rad23*	9	3	2 Identical	1
*RhoGAP102A*	5	3 (2 identical)	0	2
*Rnf11*	6	6 (4 identical)	0	0
*Slip1*	2	0	1	1
*Sox102F*	5	2 Identical	1	2
*sv*	4	0	2	2
*Syt7*	3	1	2	0
*Taf3*	3	2 Identical	1	0
*Tdg*	8	4 (2 identical)	4 (3 identical)	0
*toy*	5	4 (3 identical)	1	0
*ukar*	1	0	1	0
*yellow-h*	3	2	0	0
*zfh2*	5	5 (4 identical)	0	0
*Zyx*	3	3 Identical	0	0
**Alternative screening approach*^c^***				
*bt*	8	6 Identical	2 Identical	0
*JYalpha*	9	2	2	4
*unc-13*	4	0	1	0
*zfh2*	2	1	1	0

^*a*^In general, we sequenced target sites on mutagenized chromosomes from a screen only until we identified desirable mutations; consequently, some mutagenized chromosomes were never analyzed.

^*b*^Screens for 9 genes (*Asator*, *CamKII*, *CG32006*, *dati*, *fuss*, *Gat*, *gw*, *Mpv17*, and *PIP4 K*) using the primary screening approach produced either no mutations or no viable stocks.

^*c*^Screens for *zfh2* mutations were undertaken using both screening approaches.

^*d*^The molecular lesions associated with *Cadps^G^*, *Dyrk3^A^*, *Dyrk3^B^*, *Dyrk3^C^*, *Mitf^A^*, and *Mitf^C^* have not yet been identified.

The crosses to establish new mutations in balanced or homozygous stocks simultaneously placed the mutagenized fourth chromosomes into a defined genetic background so that, to the extent practical, the mutation-bearing stocks would differ at the sequence level only by the induced mutations. As shown in Supplementary File 1, the isogenic background consists of second and third chromosomes derived from the genetic background used for the DrosDel deletions (Ryder *et al.* 2007), the *Dp(1;Y)BSC* chromosomes (Cook *et al.* 2010) and their progenitor insertions (Ryder *et al.* 2004), and an X chromosome marked with *y^1^* and *w^1118^* derived from a heavily used Bloomington stock. The Y chromosomes in the final stocks are derived from the guide RNA stocks and cannot be assumed to be uniform. Fourth chromosomes bearing dominant mutations with visible phenotypes (e.g. *ci*^*D*^ or *Gat*^*eya*^) or transgenes expressing fluorescent reporter proteins (e.g. *P{ActGFP}unc-13^GJ^*) were used to balance the new, fourth chromosome mutations. Supplementary Table 6 lists the stocks that have been placed into public distribution to date at the Drosophila stock centers in Bloomington and Kyoto.

We encountered idiosyncratic lethality and sterility in the early crosses of 3 screens. While these problems were unrelated to the mutageneses per se and are likely attributable to cryptic mutations in the guide RNA stocks, Table 1 shows that we were able to recover mutations using the same guide RNA stocks in alternative crosses (described in Supplementary File 1). Supplementary Table 5 describes the mutations and Supplementary Table 6 lists the stocks. Note that these screens and subsequent stock constructions did not rigorously control the genetic backgrounds and that future screens by the Fourth Chromosome Resource Project will use variations of this approach and will, likewise, not control for genetic background in a disciplined way.

The predominant outcome of the mutageneses was the induction of small deletions (Supplementary Table 5). In any mutagenesis, deletions that result in frameshifted translation lead to the production of truncated proteins, while in-frame deletions lead to the loss of a small number of protein residues. Frameshifted alleles that eliminate substantial portions of proteins are likely to be amorphic, while in-frame deletion alleles may or may not affect protein function depending on the consequences of removing particular amino acids. Our goal was to recover at least 1 truncation mutation for each gene screened. We were largely successful as we recovered truncation mutations in 52 of the 72 genes for which we undertook screens (Table 1). Since the median position of the truncations was 14% of normal protein length (Supplementary Table 5), the mutations likely shorten proteins enough to result in null phenotypes. For 51 of the 52 genes with truncation mutations, all predicted protein isoforms are affected (2 of the 7 predicted *Crk* isoforms are unaffected by *Crk*^*E*^ even though, as described in the following, we showed it is a loss-of-function allele). The Fourth Chromosome Resource Project will revisit the genes for which we recovered no truncation alleles. We identified mutations in 3 genes from their lethality in complementation tests even though we have yet to identify the molecular lesions (Table 1).

While loss-of-function phenotypes have been characterized for many fourth chromosome genes, they are unknown for others. To evaluate, at least superficially, the effects of disrupting fourth chromosome genes, we assessed the viability, female fertility, and gross morphology of flies in which the new alleles were homozygous and in flies in which the new alleles were combined with preexisting mutations or chromosomal deficiencies that delete the targeted genes (the deficiencies are described in Supplementary Table 7, control crosses validating the deficiency and preexisting mutation stocks are shown in Supplementary Table 8, and the crosses evaluating the new mutations are detailed in Supplementary Table 9). Our results are summarized in Supplementary Table 10, which shows that the phenotypes of mutation homozygotes and hemizygotes are consistent. Homozygotes occasionally have more severe phenotypes than hemizygotes as one might expect if linked, deleterious mutations—preexisting or generated in the course of the crosses—were homozygosed along with the targeted mutations. Our results are presented in the context of previous phenotypic studies in Supplementary Table 11. This analysis allowed us to define the loss-of-function phenotypes for 35 genes that had never been characterized: 12 are necessary for viability while 23 have no overt loss-of-function phenotypes. It also allowed us to confirm and extend the previous characterizations of 29 genes and provide evidence that 3 in-frame deletions from our screens are loss-of-function alleles.

Several decades ago, Benjamin Hochman and colleagues attempted to determine the number of fourth chromosome loci needed for viability or female fertility via mutagenesis and complementation analyses [reviewed in Hochman (1976)]. They identified 37 loci, but they did not know how close they were to isolating mutations in all vital loci. They were aware that factors such as complex intragenic complementation patterns and the low mutability of some genes make it difficult to arrive at a definitive count of genes using their approach. Our analysis of protein-coding genes (Table 2) shows 39 genes needed for viability, 1 gene needed strictly for male fertility, 33 genes with no overt phenotypes, and 7 genes for which there is no information. We have seen no evidence that any fourth chromosome genes have phenotypes affecting only external morphology or female fertility. (The absence of genes devoted exclusively to female fertility is not surprising: Perrimon *et al*. (1986) estimated that the X chromosome, which has more than 20 times as many genes as the fourth chromosome, carries only 15 genes needed solely for female reproduction.) There is a growing consensus that noncoding RNA genes usually have subtle loss-of-function phenotypes (Liu *et al.* 2017; Schor *et al.* 2018; Camilleri-Robles *et al.* 2024). The lack of obvious phenotypes associated with deletion of the antisense RNA gene *asRNA:CR44031* (Supplementary Tables 8 and 11) and increased male-to-male courtship observed as the only apparent effect associated with deletion of the long noncoding RNA gene *lncRNA:sphinx* (Dai *et al.* 2008) are consistent with this view. If we assume that the remaining 25 noncoding RNA genes are unlikely to have lethal or sterile phenotypes, our relatively complete inventory agrees well with the count of Hochman and colleagues, suggesting that their studies were closer to identifying mutations in all vital loci than Hochman concluded in his most comprehensive review (Hochman 1976). Completion of the Fourth Chromosome Resource Project will facilitate a comprehensive assessment of loss-of-function phenotypes (including phenotypes more subtle than we assessed in our tests), and it will then be possible to determine how the Hochman vital complementation groups, most of which are still represented in extant stocks, correspond to molecularly defined genes.

**Table 2.** Loss-of-function phenotypes of the fourth chromosome, protein-coding genes.*^a^*

**Lethality**
*Actbeta, anne, apolpp, Arf4, Asator, ATPsynbeta, bt, Cadps, CamKII, CG31998, CG32006, CG33978, ci, Crk, dati, Dyrk3, eIF4G1, ey, Gat, gw, Hcf, Kif3C, lgs, MED26, Mitf, myo, onecut, pan, pho, PIP4 K, PlexA, PlexB, PMCA, RpS3A, sv, Taf3, toy, unc-13, zfh2*
**Male sterility**
*JYalpha*
**Viability**
*4E-T,Abcd1, Ank, CamKI, CG1674, CG1909, CG11360, CG32017, CG33521, CG33941, dpr7, Ekar, Eph, Ephrin, fd102C, fuss, Gyf, mav, mGluR, Mpv17, NfI, Polr1G, Pur-alpha, Rad23, RhoGAP102A, Rnf11, Sox102F, Syt7, Tdg, ukar, yellow-h, Zip102B, Zyx*
**Unknown**
*Arl4, Cals, CG31997, CG31999, CG46466^b^, ND-49, Slip1*

^*a*^ Supplementary Table 11 summarizes the evidence for categorizing each gene. We did not attempt to quantify intermediate levels of mortality. With the exception of *ey* mutations, which have well-characterized partial lethality, we categorized “lethality” as the presence of few if any viable escapers.

^*b*^New gene annotations by FlyBase scientists and collaborators added *CG46466* in 2023, changing the number of protein-coding genes from 79 to 80.

We have confirmed the presence of the basal FRT-bearing transgene *TI{TI}FRT101F* in all the final stocks by PCR amplification. Although there is no reason to suspect the mutagenesis procedure methodically corrupted the FRT-bearing transgene, we have not systematically verified that the mutation-bearing chromosomes are capable of participating in FLP-mediated mitotic recombination. Nonetheless, Stinchfield *et al*. (2024) successfully recovered mitotic clones with fourth chromosomes from 2 stocks (*Pur-alpha*^*B*^ and *zfh2*^*51A*^).

To assess whether the mutagenesis procedure produced off-target mutations on the fourth chromosome, we sampled stocks for the presence of unexpected mutations producing lethal, female-sterile, or visible phenotypes (Supplementary Table 12). We tested twelve chromosomes with targeted mutations that were lethal (*Actbeta*^*B*^, *apolpp*^*B*^, *Arf4^A^*, *ATPsynbeta*^*C*^, *bt*^*A*^, *CG31998*^*C*^, *CG31998*^*D*^, *CG33978*^*B*^*, ci*^*C*^, *ey*^*B*^, *pho*^*F*^, *sv*^*F*^, *toy*^*C*^) for the presence of additional deleterious mutations by combining these chromosomes via crosses with a panel of deficiencies and mutations that together delete or disrupt all fourth chromosome genes. None of these chromosomes showed evidence of additional mutations. Similarly, we tested 5 chromosomes with targeted mutations that produced no overt phenotypes in combination with deficiencies or preexisting mutations yet showed appreciable recessive lethality or sterility in stocks (*CG32017*^*K*^, *Gyf*^*F*^, *Hcf*^*A*^, *Pur-alpha*^*B*^, *Slip1*^*A*^). None of these chromosomes had additional mutations. The poor viability in these and similar stocks may come from sublethal effects of the targeted mutations combined with sublethal effects of other mutations that were induced during the mutagenesis or that arose spontaneously in subsequent culture. The conclusions we can draw from all these crosses are limited: we did not test all mutated chromosomes, and it was impossible to test for off-target mutations in every fourth chromosome gene because some genes are deleted only by the same deficiencies as targeted genes. Nevertheless, these results indicate that off-target mutations were not common.

In summary, the work described here has provided a new resource for examining the cellular functions of most protein-coding genes on the fourth chromosome. It complements the other resources being generated by the Fourth Chromosome Resource Project (Stinchfield *et al.* 2024) including *UAS* constructs for fourth chromosome gene misexpression and phenotypic rescue, *GAL4* knock-ins for expressing *UAS* constructs in the normal patterns of fourth chromosome genes, *UAS* constructs for the human homologs of Drosophila fourth chromosome genes for creating “humanized” flies with human gene expression substituted for fly gene expression, *GFP* knock-ins into fourth chromosome genes for expressing fluor-tagged proteins in native gene patterns, and *UAS-RNAi* transgenes for GAL4-directed, tissue-specific knockdown of fourth chromosome genes. Our hope is that these resources will alleviate many of the difficulties that have historically hampered investigations of fourth chromosome gene functions.

## Supplementary Material

jkaf006_Supplementary_Data

## Data Availability

The accompanying tables contain complete mutation-characterization data. Stocks for mutations chosen for public distribution may be obtained from the Bloomington Drosophila Stock Center or the Department of Drosophila Genomics and Genetics Resources of the Kyoto Institute of Technology as indicated in Supplementary Table 6. Supplemental material available at G3 online.
