# Supplementary material for: A new Drosophila melanogaster research resource: CRISPR-induced mutations for clonal analysis of fourth chromosome genes
Source: G3 (Bethesda). 2025 Jan 13;15(3):jkaf006. doi: 10.1093/g3journal/jkaf006 (PMC11917476; doi:10.1093/g3journal/jkaf006)
Supplement: jkaf006_Supplementary_Data [file jkaf006_supplementary_data.zip › Supplementary_File_1_G3-2024-405535.docx]

**Supplementary File 1. Mutagenesis and mutation characterization**

**Primary approach for CRISPR mutagenesis and the construction of isogenic stocks**

The following genetic scheme shows how we generated mutations on an isogenic, FRT-bearing fourth chromosome in the male germ line using guide RNA constructs located on the second chromosome, and how we established the mutations in stocks sharing a defined genetic background. The guide RNA constructs (Supplementary Table 1) are shown generically as *P{U6-gRNA}* to indicate that the guide RNA was under the control of *snRNA:U6:96Ab* regulatory sequences.

G0: $\frac{y^{1}w^{*}}{y^{1}w^{*}}; \frac{P\left\{ w^{+mC}=EP \right\}MESR3^{G19459}}{CyO}; \frac{P\left\{ w^{+mC}=ActGFP \right\}unc\text{-}{13}^{GJ}}{+}$ ♀♀ x $\frac{y^{2}cho^{1}v^{1}}{Y}; \frac{P\left\{ y^{+t7.7}v^{+t1.8}=U6\text{-}gRNA \right\}attP40}{P\left\{ y^{+t7.7}v^{+t1.8}=U6\text{-}gRNA \right\}attP40}$ ♂♂

G1: $\frac{y^{1}w^{*}}{y^{1}w^{*}}; \frac{P\left\{ y^{+t7.7}v^{+t1.8}=nanos\text{-}Cas9.R \right\}attP40}{CyO}; \frac{TI\left\{ TI \right\}FRT101F iso}{TI\left\{ TI \right\}FRT101F iso}$ ♀♀

x $\frac{y^{1}w^{*}}{Y}; \frac{CyO}{P\left\{ y^{+t7.7}v^{+t1.8}=U6\text{-}gRNA \right\}attP40}; \frac{P\left\{ w^{+mC}=ActGFP \right\}unc\text{-}{13}^{GJ}}{+}$ ♂♂

G2: $\frac{y^{1}w^{*}}{y^{1} w^{*}}; \frac{P\left\{ w^{+mC}=EP \right\}G16942}{P\left\{ w^{+mC}=EP \right\}G16942}; \frac{M\left\{ 3xP3\text{-}RFP.attP \right\}ZH\text{-}102D}{M\left\{ 3xP3\text{-}RFP.attP \right\}ZH\text{-}102D}$ ♀♀

x $\frac{y^{1}w^{*}}{Y}; \frac{P\left\{ y^{+t7.7}v^{+t1.8}=nanos\text{-}Cas9.R \right\}attP40}{P\left\{ y^{+t7.7}v^{+t1.8}=U6\text{-}gRNA \right\}attP40}; \frac{TI\left\{ TI \right\}FRT101F iso}{P\left\{ w^{+mC}=ActGFP \right\}unc\text{-}{13}^{GJ}}$ ♂♂

G3: $\frac{y^{1}w^{1118}iso}{y^{1}w^{1118}iso}; \frac{iso2}{iso2}; \frac{iso3}{iso3}; \frac{P\left\{ w^{+mC}=ActGFP \right\}unc\text{-}{13}^{GJ}}{In\left( 4 \right)ci^{\text{D }}\text{,} ci^{D}pan^{ciD}}$ ♀♀

x $\frac{y^{1}w^{*}}{Y}; \frac{\text{+}}{\left( P\left\{ y^{+t7.7}v^{+t1.8}=nanos\text{-}Cas9.R \right\}attP40 \mathrm{or} P\left\{ y^{+t7.7}v^{+t1.8}=U6\text{-}gRNA \right\}attP40 \right)}$;

$\frac{P\left\{ w^{+mC}=EP \right\}G16942}{\text{+}}; \frac{M\left\{ 3xP3\text{-}RFP.attP \right\}ZH\text{-}102D}{TI\left\{ TI \right\}FRT101F mutation}$ single ♂

G4: $\frac{y^{1}w^{1118}iso}{y^{1}w^{1118}iso}; \frac{iso2}{iso2}; \frac{iso3}{iso3}; \frac{P\left\{ w^{+mC}=ActGFP \right\}unc\text{-}{13}^{GJ}}{In\left( 4 \right)ci^{D}\text{,} ci^{D}pan^{ciD}}$ ♀♀

x $\frac{y^{1}w^{1118}iso}{Y}; \frac{iso2}{(P\left\{ y^{+t7.7}v^{+t1.8}=nanos\text{-}Cas9.R \right\}attP40\text{ or}\text{ }\text{P}\left\{ y^{+t7.7}v^{+t1.8}=U6\text{-}gRNA \right\}attP40)}$;

$\frac{iso3}{P\left\{ w^{+mC}=EP \right\}G16942}; \frac{In\left( 4 \right)ci^{D}\text{,} ci^{D}pan^{ciD}}{TI\left\{ TI \right\}FRT101F mutation}$ ♂♂

G5: $\frac{y^{1}w^{1118}iso}{y^{1}w^{1118}iso}; \frac{iso2}{iso2}; \frac{iso3}{iso3}; \frac{P\left\{ w^{+mC}=ActGFP \right\}unc\text{-}{13}^{GJ}}{In\left( 4 \right)ci^{D}\text{,} ci^{D}pan^{ciD}}$ ♀♀

x $\frac{y^{1}w^{1118}iso}{Y}; \frac{iso2}{iso2}; \frac{iso3}{iso3}; \frac{In\left( 4 \right)ci^{D}\text{,} ci^{D}pan^{ciD}}{TI\left\{ TI \right\}FRT101F mutation}$ ♂♂

G6: $\frac{y^{1}w^{1118}iso}{y^{1}w^{1118}iso}; \frac{iso2}{iso2}; \frac{iso3}{iso3}; \frac{In\left( 4 \right)ci^{D}\text{,} ci^{D}pan^{ciD}}{TI\left\{ TI \right\}FRT101F mutation}$ ♀♀ x $\frac{y^{1}w^{1118}iso}{Y}; \frac{iso2}{iso2}; \frac{iso3}{iso3}; \frac{In\left( 4 \right)ci^{D}\text{,} ci^{D}pan^{ciD}}{TI\left\{ TI \right\}FRT101F mutation}$ ♂♂

Note that in G1 we introduced a target FRT-bearing fourth chromosome that had been isogenized in crosses we will show below. This assured, as much as possible, that all mutation-bearing chromosomes have the same sequence other than the mutation at the CRISPR target site. After isolation of the mutation-bearing fourth chromosomes in G3, they were crossed into a genetic background with defined X, second and third chromosomes (indicated as “iso” in the crosses). In G3, multiple single-male crosses established independent lines, which were carried through the remaining crosses. The CRISPR target sites were sequenced for each line as described below. Typically, few than 10 lines were established for each set of crosses involving a particular guide RNA construct. If the progeny of a G6 cross were viable and fertile, we established a homozygous stock.

Similarly, the following crosses show how we screened for mutations using guide RNA constructs on the third chromosome.

G0: $\frac{y^{1}w^{*}}{y^{1}w^{*}}; \frac{P\left\{ w^{+mC}=EP \right\}G16942}{P\left\{ w^{+mC}=EP \right\}G16942}; \frac{In\left( 4 \right)ciD\text{, }\text{ci}^{D}pan^{ciD}}{+}$ ♀♀ x $\frac{y^{2}cho^{1}v^{1}}{Y}; \frac{P\left\{ y^{+t7.7}v^{+t1.8}=U6\text{-}gRNA \right\}attP2}{P\left\{ y^{+t7.7}v^{+t1.8}=U6\text{-}gRNA \right\}attP2}$ ♂♂

G1: $\frac{y^{1}w^{*}}{y^{1}w^{*}}; \frac{P\left\{ y^{+t7.7}v^{+t1.8}=nos\text{-}Cas9.R \right\}attP2}{P\left\{ y^{+t7.7}v^{+t1.8}=nos\text{-}Cas9.R \right\}attP2}; \frac{TI\left\{ TI \right\}FRT101F iso}{TI\left\{ TI \right\}FRT101F iso}$ ♀♀

x $\frac{y^{1}w^{*}}{Y}; \frac{P\left\{ y^{+mDint2}w^{+mC}=EPgy2 \right\}CG{17801}^{EY09613}}{P\left\{ y^{+t7.7}v^{+t1.8}=U6\text{-}gRNA \right\}attP2}; \frac{In\left( 4 \right)ci^{D}\text{,} ci^{D}pan^{ciD}}{\text{+}}$ ♂♂

G2: $\frac{y^{1}w^{*}}{y^{1}w^{*}}; \frac{P\left\{ w^{+mC}=EP \right\}MESR3^{G19459}}{P\left\{ w^{+mC}=EP \right\}MESR3^{G19459}}; \frac{M\left\{ 3xP3\text{-}RFP.attP \right\}ZH\text{-}102D}{M\left\{ 3xP3\text{-}RFP.attP \right\}ZH\text{-}102D}$ ♀♀

x $\frac{y^{1}w^{*}}{Y}; \frac{P\left\{ y^{+t7.7}v^{+t1.8}=nanos\text{-}Cas9.R \right\}attP2}{P\left\{ y^{+t7.7}v^{+t1.8}=U6\text{-}gRNA \right\}attP2}; \frac{TI\left\{ TI \right\}FRT101F iso}{In\left( 4 \right)ci^{D}\text{,} ci^{D}pan^{ciD}}$ ♂♂

G3: $\frac{y^{1}w^{1118}iso}{y^{1}w^{1118}iso}; \frac{iso2}{iso2}; \frac{iso3}{iso3}; \frac{P\left\{ w^{+mC}=ActGFP \right\}unc\text{-}{13}^{GJ}}{In\left( 4 \right)ci^{D}\text{,} ci^{D}pan^{ciD}}$ ♀♀

x $\frac{y^{1}w^{*}}{Y}; \frac{P\left\{ w^{+mC}=EP \right\}MESR3^{G19459}}{+}; \frac{\text{+}}{(P\left\{ y^{+t7.7}v^{+t1.8}=nanos\text{-}Cas9.R \right\}attP2 \text{or} P\left\{ y^{+t7.7}v^{+t1.8}=U6\text{-}gRNA \right\}attP2)}$;

$\frac{M\left\{ 3xP3\text{-}RFP.attP \right\}ZH\text{-}102D}{TI\left\{ TI \right\}FRT101F mutation}$ single ♂

G4: $\frac{y^{1}w^{1118}iso}{y^{1}w^{1118}iso}; \frac{iso2}{iso2}; \frac{iso3}{iso3}; \frac{P\left\{ w^{+mC}=ActGFP \right\}unc\text{-}{13}^{GJ}}{In\left( 4 \right)ci^{D}\text{,} ci^{D}pan^{ciD}}$ ♀♀

x $\frac{y^{1}w^{1118}iso}{Y}; \frac{iso2}{P\left\{ w^{+mC}=EP \right\}MESR3^{G19459}}; \frac{iso3}{(P\left\{ y^{+t7.7}v^{+t1.8}=nanos\text{-}Cas9.R \right\}attP2 \text{or} P\left\{ y^{+t7.7}v^{+t1.8}=U6\text{-}gRNA \right\}attP2)}$;

$\frac{In\left( 4 \right)ci^{D}\text{,} ci^{D}pan^{ciD}}{TI\left\{ TI \right\}FRT101F mutation}$ ♂♂

G5: $\frac{y^{1}w^{1118}iso}{y^{1}w^{1118}iso}; \frac{iso2}{iso2}; \frac{iso3}{iso3}; \frac{P\left\{ w^{+mC}=ActGFP \right\}unc\text{-}{13}^{GJ}}{In\left( 4 \right)ci^{D}\text{, }ci^{D}pan^{ciD}}$ ♀♀ x $\frac{y^{1}w^{1118}iso}{Y}; \frac{iso2}{iso2}; \frac{iso3}{iso3}; \frac{In\left( 4 \right)ci^{D}\text{,} ci^{D}pan^{ciD}}{TI\left\{ TI \right\}FRT101F mutation}$ ♂♂

G6: $\frac{y^{1}w^{1118}iso}{y^{1}w^{1118}iso}; \frac{iso2}{iso2}; \frac{iso3}{iso3}; \frac{In\left( 4 \right)ci^{D}\text{,} ci^{D}pan^{ciD}}{TI\left\{ TI \right\}FRT101F mutation}$ ♀♀ x $\frac{y^{1}w^{1118}iso}{Y}; \frac{iso2}{iso2}; \frac{iso3}{iso3}; \frac{In\left( 4 \right)ci^{D}\text{,} ci^{D}pan^{ciD}}{TI\left\{ TI \right\}FRT101F mutation}$ ♂♂

Each of these modes of screening for fourth chromosome mutations required us to generate stocks beforehand. The following genetic scheme shows how we generated stocks for use in G3, G4 and G5 of both of the screening schemes above with defined X, second and third chromosomes and fourth chromosomes carrying dominant markers. The X chromosome was derived from a stock with a commonly used *y^1^ w^1118^* chromosome. The second and third chromosomes are derived from stocks that were constructed by Ryder *et al*. (Ryder *et al.* 2004) and Cook *et al*. (Cook *et al.* 2010) to have various chromosomes introgressed into a common isogenic background. This assures that the second and third chromosomes in the final fourth chromosome mutation stocks have minimal genetic variability, containing only those polymorphisms that may have arisen since the progenitor stocks (6878, 35531 and 35332) were established. The numbers here and below refer to Bloomington Stock Center stock numbers.

G0: 950 $\frac{Df\left( 1 \right)RA2}{FM7c}$ ♀♀ x 9549 $\frac{w^{1118}}{w^{1118}}; \frac{P\left\{ w^{+mC}=ActGFP \right\}unc\text{-}{13}^{GJ}}{In\left( 4 \right)ci^{D}\text{, }ci^{D}pan^{ciD}}$ ♂♂ 117 $\frac{C\left( 1 \right)DX, y^{1}f^{1}}{Y}$ ♀♀ x 6598 $\frac{y^{1}w^{1118}}{Y}$ single ♂

G1: $\frac{FM7c}{w^{1118}}; \frac{\text{+}}{(P\left\{ w^{+mC}=ActGFP \right\}unc\text{-}{13}^{GJ}\text{or} In\left( 4 \right)ci^{D}\text{,} ci^{D}pan^{ciD})}$ ♀♀ x $\frac{y^{1}w^{1118}iso}{Y}$ single ♂

17599 $\frac{y^{1}w^{67c23}}{y^{1}w^{67c23}}; \frac{P\left\{ w^{+mC}=EPgy2 \right\}EY09758}{P\left\{ w^{+mC}=EPgy2 \right\}EY09758}$ ♀♀ x 525 $\frac{Ki^{1}}{Ki^{1}}$ ♂♂

G2: $\frac{y^{1}w^{1118}iso}{FM7c}; \frac{\text{+}}{(P\left\{ w^{+mC}=ActGFP \right\}unc\text{-}{13}^{GJ}\text{or} In\left( 4 \right)ciD\text{, } ci^{D}pan^{ciD})}$ ♀♀ x $\frac{y^{1}w^{67c23}}{Y}; \frac{P\left\{ w^{+mC}=EPgy2 \right\}EY09758}{\text{+}}; \frac{\text{+}}{Ki^{1}}$ ♂♂

G3: 35332 $\frac{C\left( 1 \right)RA\text{,} In\left( 1 \right)sc^{J1}\text{,} In\left( 1 \right)sc^{8}\text{,} l\left( 1 \right)1Ac^{1}\text{,} sc^{J1}sc^{8}}{Dp\left( 1;Y \right)y^{\text{+}}}; \frac{iso2}{iso2}; \frac{iso3}{iso3}$ ♀♀

x $\frac{y^{1}w^{1118}iso}{Y}; \frac{\text{+}}{P\left\{ w^{+mC}=EPgy2 \right\}EY09758}; \frac{\text{+}}{Ki^{1}}; \frac{\text{+}}{\left( P\left\{ w^{+mC}=ActGFP \right\}unc\text{-}{13}^{GJ}\text{or} In\left( 4 \right)ci^{D}\text{,} ci^{D}pan^{ciD} \right)}$ ♂♂

G4: 6878 $\frac{P\left\{ w^{+mW.Scer\backslash FRT.hs}=RS3 \right\}l\left( 1 \right)CG\text{-}6411\text{-}3^{1}, w^{1118}}{FM7h}; \frac{iso2}{iso2}; \frac{iso3}{iso3}$ ♀♀

x $\frac{y^{1}w^{1118}iso}{Y}; \frac{iso2}{P\left\{ w^{+mC}=EPgy2 \right\}EY09758}; \frac{iso3}{Ki^{1}}; \frac{\text{+}}{(P\left\{ w^{+mC}=ActGFP \right\}unc\text{-}{13}^{GJ}\text{or} In\left( 4 \right)ci^{D}\text{,} ci^{D}pan^{ciD})}$ ♂♂

G5: $\frac{FM7h}{y^{1}w^{1118}iso}; \frac{iso2}{iso2}; \frac{iso3}{iso3}; \frac{\text{+}}{(P\left\{ w^{+mC}=ActGFP \right\}unc\text{-}{13}^{GJ}or In\left( 4 \right)ci^{D}\text{,} ci^{D}pan^{ciD})}$ ♀♀

x 35531 $\frac{winscy}{Dp\left( 2:Y \right)G\text{,} P\left\{ w^{+mC}=hs\text{-}hid \right\}Y}; \frac{iso2}{iso2}; \frac{iso3}{iso3}$ ♂♂

G6: $\frac{FM7h}{y^{1}w^{1118}iso}; \frac{iso2}{iso2}; \frac{iso3}{iso3}; \frac{\text{+}}{(P\left\{ w^{+mC}=ActGFP \right\}unc\text{-}{13}^{GJ}or In\left( 4 \right)ci^{D}\text{,} ci^{D}pan^{ciD})}$ ♀♀

x $\frac{y^{1}w^{1118}iso}{Dp\left( 2:Y \right)G\text{,} P\left\{ w^{+mC}=hs\text{-}hid \right\}Y}; \frac{iso2}{iso2}; \frac{iso3}{iso3}; \frac{P\left\{ w^{+mC}=ActGFP \right\}unc\text{-}{13}^{GJ}}{In\left( 4 \right)ci^{D}\text{,} ci^{D}pan^{ciD}}$ ♂♂

G7: $\frac{y^{1}w^{1118}iso}{y^{1}w^{1118}iso}; \frac{iso2}{iso2}; \frac{iso3}{iso3}; \frac{P\left\{ w^{+mC}=ActGFP \right\}unc\text{-}{13}^{GJ}}{In\left( 4 \right)ci^{D}\text{,} ci^{D}pan^{ciD}}$ ♀♀

x $\frac{y^{1}w^{1118}iso}{Dp\left( 2:Y \right)G\text{,} P\left\{ w^{+mC}=hs\text{-}hid \right\}Y}; \frac{iso2}{iso2}; \frac{iso3}{iso3}; \frac{P\left\{ w^{+mC}=ActGFP \right\}unc\text{-}{13}^{GJ}}{In\left( 4 \right)ci^{D}\text{,} ci^{D}pan^{ciD}}$ ♂♂

Here we will detail the stock constructions needed for screens involving second chromosome insertions of guide RNA constructs.

The following genetic scheme shows how we generated the stock used in G1 with an isogenic *FRT*-bearing fourth chromosome and a second chromosome *nanos-Cas9* insertion.

G0: 24800 $\frac{y^{1}w^{67c23}}{y^{1}w^{67c23}}; \frac{Mi\left\{ GFP^{E\text{.}3xP3}=ET1 \right\}CG{32017}^{MB02477}}{Mi\left\{ GFP^{E\text{.}3xP3}=ET1 \right\}CG{32017}^{MB02477}}$ ♀♀

x 78781 $\frac{y^{1}sc^{*}v^{1}sev^{21}}{Y}; \frac{P\left\{ y^{+t7.7}v^{+t1.8}=nanos\text{-}Cas9.R \right\}attP40}{CyO}$ ♂♂

G1: $\frac{y^{1}w^{67c23}}{y^{1}sc^{*}v^{1}sev^{21}}; \frac{\text{+}}{CyO}$ ; $\frac{Mi\left\{ GFP^{E\text{.}3xP3}=ET1 \right\}CG{32017}^{MB02477}}{\text{+}}$ ♀♀

x $\frac{y^{1}w^{67c23}}{Y}; \frac{\text{+}}{P\left\{ y^{+t7.7}v^{+t1.8}=nanos\text{-}Cas9.R \right\}attP40}; \frac{Mi\left\{ GFP^{E. 3xP3}=ET1 \right\}CG{32017}^{MB02477}}{\text{+}}$ ♂♂

G2: $\frac{y^{1}w^{67c23}}{y^{1}w^{67c23}}; \frac{CyO}{P\left\{ y^{+t7.7}v^{+t1.8}=nanos\text{-}Cas9.R \right\}attP40}; \frac{Mi\left\{ GFP^{E\text{.}3xP3}=ET1 \right\}CG{32017}^{MB02477}}{(Mi\left\{ GFP^{E\text{.}3xP3}=ET1 \right\}CG{32017}^{MB02477}\text{or} \text{+)}}$ ♀♀

x $\frac{y^{1}w^{67c23}}{Y}; \frac{P\left\{ y^{+t7.7}v^{+t1.8}=nanos\text{-}Cas9.R \right\}attP40}{CyO}; \frac{Mi\left\{ GFP^{E\text{.}3xP3}=ET1 \right\}CG{32017}^{MB02477}}{(Mi\left\{ GFP^{E\text{.}3xP3}=ET1 \right\}CG{32017}^{MB02477}\text{or} \text{+)}}$ ♂♂

Maintained as selected stock until fourth chromosome went homozygous and then used in following cross

G3: $\frac{y^{1}w^{67c23}}{y^{1}w^{67c23}}; \frac{P\left\{ y^{+t7.7}v^{+t1.8}=nanos\text{-}Cas9.R \right\}attP40}{CyO}; \frac{Mi\left\{ GFP^{E\text{.}3xP3}=ET1 \right\}CG{32017}^{MB02477}}{Mi\left\{ GFP^{E\text{.}3xP3}=ET1 \right\}CG{32017}^{MB02477}}$ ♀♀ x $\frac{y^{1}w^{*}}{Y}; \frac{TI\left\{ TI \right\}FRT101F iso}{TI\left\{ TI \right\}FRT101F iso}$ ♂♂

G4: $\frac{y^{1}w^{67c23}}{y^{1}w^{1118}}; \frac{CyO}{+}; \frac{Mi\left\{ GFP^{E\text{.}3xP3}=ET1 \right\}CG{32017}^{MB02477}}{TI\{TI\}FRT101F iso}$ ♀♀

x $\frac{y^{1}w^{67c23}}{Y}; \frac{P\left\{ y^{+t7.7}v^{+t1.8}=nanos\text{-}Cas9.R \right\}attP40}{\text{+}}; \frac{TI\left\{ TI \right\}FRT101F iso}{Mi\left\{ GFP^{E\text{.}3xP3}=ET1 \right\}CG{32017}^{MB02477}}$ ♂♂

G5: $\frac{y^{1}w^{*}}{y^{1}w^{*}}; \frac{CyO}{P\left\{ y^{+t7.7}v^{+t1.8}=nanos\text{-}Cas9.R \right\}attP40}; \frac{TI\left\{ TI \right\}FRT101F iso}{TI\left\{ TI \right\}FRT101F iso}$ ♀♀

x $\frac{y^{1}w^{*}}{Y}; \frac{CyO}{P\left\{ y^{+t7.7}v^{+t1.8}=nanos\text{-}Cas9.R \right\}attP40}; \frac{TI\left\{ TI \right\}FRT101F iso}{TI\left\{ TI \right\}FRT101F iso}$ ♂♂

The following crosses show how we generated the stock used in G3 with an isogenic, FRT-bearing fourth chromosome.

G0: 9549 $\frac{w^{1118}}{w^{1118}}; \frac{P\left\{ w^{+mC}=ActGFP \right\}unc\text{-}{13}^{GJ}}{In\left( 4 \right)ci^{D}\text{,} ci^{D}pan^{ciD}}$ ♀♀ x 94596 $\frac{w^{1118}}{Y}; \frac{TI\left\{ TI \right\}FRT101F}{TI\left\{ TI \right\}FRT101F}$ single ♂

G1: 9549 $\frac{w^{1118}}{w^{1118}}; \frac{P\left\{ w^{+mC}=ActGFP \right\}unc\text{-}{13}^{GJ}}{In\left( 4 \right)ci^{D}\text{,} ci^{D}pan^{ciD}}$ ♀♀ x $\frac{w^{1118}}{Y}; \frac{TI\left\{ TI \right\}FRT101F}{In\left( 4 \right)ci^{D}\text{,} ci^{D}pan^{ciD}}$ single ♂

G2: $\frac{w^{1118}}{w^{1118}}; \frac{TI\left\{ TI \right\}FRT101F iso}{In\left( 4 \right)ci^{D}\text{,} ci^{D}pan^{ciD}}$ ♀♀ x $\frac{w^{1118}}{Y}; \frac{TI\left\{ TI \right\}FRT101F iso}{In\left( 4 \right)ci^{D}\text{,} ci^{D}pan^{ciD}}$ ♂♂

G3: $\frac{w^{1118}}{w^{1118}}; \frac{TI\left\{ TI \right\}FRT101F iso}{TI\left\{ TI \right\}FRT101F iso}$ ♀♀ x $\frac{w^{1118}}{Y}; \frac{TI\left\{ TI \right\}FRT101F iso}{TI\left\{ TI \right\}FRT101F iso}$ ♂♂

Maintained as a stock and used in following cross

G4: $\frac{y^{1}w^{1118}iso}{y^{1}w^{1118}iso}; \frac{iso2}{iso2}; \frac{iso3}{iso3}; \frac{P\left\{ w^{+mC}=ActGFP \right\}unc\text{-}{13}^{GJ}}{In\left( 4 \right)ci^{D}\text{,} ci^{D}pan^{ciD}}$ ♀♀ x $\frac{w^{1118}}{Y}; \frac{TI\left\{ TI \right\}FRT101F iso}{TI\left\{ TI \right\}FRT101F iso}$ ♂♂

G5: $\frac{y^{1}w^{1118}iso}{w^{1118}}; \frac{TI\left\{ TI \right\}FRT101F iso}{In\left( 4 \right)ci^{D}\text{,} ci^{D}pan^{ciD}}$ ♀♀ x $\frac{y^{1}w^{1118}iso}{Y}; \frac{TI\left\{ TI \right\}FRT101F iso}{In\left( 4 \right)ci^{D}\text{,} ci^{D}pan^{ciD}}$ ♂♂

G6: $\frac{y^{1}w^{*}}{y^{1}w^{*}}; \frac{TI\left\{ TI \right\}FRT101F iso}{TI\left\{ TI \right\}FRT101F iso}$ ♀♀ x $\frac{y^{1}w^{*}}{Y}; \frac{TI\left\{ TI \right\}FRT101F iso}{TI\left\{ TI \right\}FRT101F iso}$ ♂♂

The following genetic scheme shows how we generated the stock used in G0 of the screen with a miniwhite-marked transgene insertion on the second chromosome (chosen simply for the visible marker and the vigor of the stock) and a dominant fourth chromosome marker on the fourth chromosome.

GO: 9549 $\frac{w^{1118}}{w^{1118}}; \frac{P\left\{ w^{+mC}=ActGFP \right\}unc\text{-}{13}^{GJ}}{In\left( 4 \right)ci^{D}\text{,} ci^{D}pan^{ciD}}$ ♀♀ x 24639 $\frac{w^{1118}}{Dp\left( 2:Y \right)G\text{,} P\left\{ w^{+mC}=hs\text{-}hid \right\}Y}; \frac{wg^{Sp\text{-}1}}{CyO}$ ♂♂

G1: 26964 $\frac{y^{1}w^{*}}{y^{1}w^{*}}; \frac{P\left\{ w^{+mC}=EP \right\}MESR3^{G19459}}{P\left\{ w^{+mC}=EP \right\}MESR3^{G19459}}$ ♀♀ x $\frac{w^{1118}}{Dp\left( 2:Y \right)G\text{,} P\left\{ w^{+mC}=hs\text{-}hid \right\}Y}; \frac{\text{+}}{CyO}; \frac{P\left\{ w^{+mC}=ActGFP \right\}unc\text{-}{13}^{GJ}}{\text{+}}$ ♂♂

G2: $\frac{y^{1}w^{*}}{w^{1118}}; \frac{P\left\{ w^{+mC}=EP \right\}MESR3^{G19459}}{CyO}; \frac{\text{+}}{P\left\{ w^{+mC}=ActGFP \right\}unc\text{-}{13}^{GJ}}$ ♀♀

x $\frac{w^{1118}}{Dp\left( 2:Y \right)G\text{,} P\left\{ w^{+mC}=hs\text{-}hid \right\}Y}; \frac{P\left\{ w^{+mC}=EP \right\}MESR3^{G19459}}{CyO}; \frac{\text{+}}{P\left\{ w^{+mC}=ActGFP \right\}unc\text{-}{13}^{GJ}}$ ♂♂

Selected stock for y^–^ and GFP

The following crosses generated the stock used in G2 of the screen with a homozygous viable and fertile, miniwhite-marked, third chromosome insertion (chosen for the visible marker and the vigor of the stock) and a fourth chromosome insertion expressing RFP.

G0: 24488 $\frac{y^{1}M\left\{ RFP^{3xP3\text{.}PB}GFP^{E\text{.}3xP3}=vas\text{-}int.Dm \right\}ZH\text{-}2A w^{*}}{y^{1}M\left\{ RFP^{3xP3\text{.}PB}GFP^{E\text{.}3xP3}=vas\text{-}int.Dm \right\}ZH\text{-}2A w^{*}}; \frac{M\left\{ 3xP3\text{-}RFP.attP \right\}ZH\text{-}102D}{M\left\{ 3xP3\text{-}RFP.attP \right\}ZH\text{-}102D}$ ♀♀

x 24640 $\frac{w^{1118}}{Dp\left( 2:Y \right)G\text{,} P\left\{ w^{+mC}=hs\text{-}hid \right\}Y}; \frac{MKRS}{TM2\text{,} y^{\text{+}}}$ ♂♂

G1: 26932 $\frac{y^{1}w^{*}}{y^{1}w^{*}}; \frac{P\left\{ w^{+mC}=EP \right\}G16942}{P\left\{ w^{+mC}=EP \right\}G16942}$ ♀♀

x $\frac{y^{1}M\left\{ RFP^{3xP3\text{.}PB}GFP^{E\text{.}3xP3}=vas\text{-}int.Dm \right\}ZH\text{-}2A w^{*}}{Dp\left( 2:Y \right)G\text{,} P\left\{ w^{+mC}=hs\text{-}hid \right\}Y}; \frac{\text{+}}{MKRS}; \frac{M\left\{ 3xP3\text{-}RFP.attP \right\}ZH\text{-}102D}{\text{+}}$ ♂♂

G2: 26932 $\frac{y^{1}w^{*}}{y^{1}w^{*}}; \frac{P\left\{ w^{+mC}=EP \right\}G16942}{P\left\{ w^{+mC}=EP \right\}G16942}$ ♀♀

x $\frac{y^{1}w^{*}}{Dp\left( 2:Y \right)G\text{,} P\left\{ w^{+mC}=hs\text{-}hid \right\}Y}; \frac{P\left\{ w^{+mC}=EP \right\}G16942}{MKRS}; \frac{\text{+}}{M\left\{ 3xP3\text{-}RFP.attP \right\}ZH\text{-}102D}$ ♂♂

G3: $\frac{y^{1}w^{*}}{y^{1}w^{*}}; \frac{P\left\{ w^{+mC}=EP \right\}G16942}{P\left\{ w^{+mC}=EP \right\}G16942}; \frac{M\left\{ 3xP3\text{-}RFP.attP \right\}ZH\text{-}102D}{\text{+}}$ ♀♀

x $\frac{y^{1}w^{*}}{Dp\left( 2:Y \right)G\text{,} P\left\{ w^{+mC}=hs\text{-}hid \right\}Y}; \frac{P\left\{ w^{+mC}=EP \right\}G16942}{P\left\{ w^{+mC}=EP \right\}G16942}; \frac{M\left\{ 3xP3\text{-}RFP.attP \right\}ZH\text{-}102D}{\text{+}}$ ♂♂

G4: $\frac{y^{1}w^{*}}{y^{1}w^{*}}; \frac{P\left\{ w^{+mC}=EP \right\}G16942}{P\left\{ w^{+mC}=EP \right\}G16942}; \frac{M\left\{ 3xP3\text{-}RFP.attP \right\}ZH\text{-}102D}{M\left\{ 3xP3\text{-}RFP.attP \right\}ZH\text{-}102D}$ ♀♀

x $\frac{y^{1}w^{*}}{Dp\left( 2:Y \right)G\text{,} P\left\{ w^{+mC}=hs\text{-}hid \right\}Y}; \frac{P\left\{ w^{+mC}=EP \right\}G16942}{P\left\{ w^{+mC}=EP \right\}G16942}; \frac{M\left\{ 3xP3\text{-}RFP\text{.}attP \right\}ZH\text{-}102D}{M\left\{ 3xP3\text{-}RFP\text{.}attP \right\}ZH\text{-}102D}$ ♂♂

Selected stock until no non-RFP progeny appeared

Here we will detail the stock constructions needed for screens involving third chromosome insertions of guide RNA constructs. The stock generated in the genetic scheme shown immediately above was also used in these screens.

The following genetic scheme shows how we generated the stock used in G1 of the screen with an isogenic *FRT*-bearing fourth chromosome and a third chromosome *nanos-Cas9* insertion.

G0: 24800 $\frac{y^{1}w^{67c23}}{y^{1}w^{67c23}}; \frac{Mi\left\{ GFP^{E\text{.}3xP3}=ET1 \right\}CG{32017}^{MB02477}}{Mi\left\{ GFP^{E\text{.}3xP3}=ET1 \right\}CG{32017}^{MB02477}}$ ♀♀

x 78782 $\frac{y^{1}sc^{*}v^{1}sev^{21}}{Y}; \frac{P\left\{ y^{+t7.7}v^{+t1.8}=nanos\text{-}Cas9\text{.}R \right\}attP2}{P\left\{ y^{+t7.7}v^{+t1.8}=nanos\text{-}Cas9\text{.}R \right\}attP2}$ ♂♂

G1: $\frac{y^{1}w^{67c23}}{y^{1}sc^{*}v^{1}sev^{21}}; \frac{\text{+}}{P\left\{ y^{+t7.7}v^{+t1.8}=nanos\text{-}Cas9.R \right\}attP2}; \frac{Mi\left\{ GFP^{E\text{.}3xP3}=ET1 \right\}CG{32017}^{MB02477}}{\text{+}}$ ♀♀

x $\frac{y^{1}w^{67c23}}{Y}; \frac{\text{+}}{P\left\{ y^{+t7.7}v^{+t1.8}=nanos\text{-}Cas9.R \right\}attP2}; \frac{Mi\left\{ GFP^{E\text{.}3xP3}=ET1 \right\}CG{32017}^{MB02477}}{\text{+}}$ ♂♂

G2: $\frac{y^{1}w^{67c23}}{y^{1}w^{67c23}}; \frac{P\left\{ y^{+t7.7}v^{+t1.8}=nanos\text{-}Cas9.R \right\}attP2}{(P\left\{ y^{+t7.7}v^{+t1.8}=nanos\text{-}Cas9.R \right\}attP2 \text{or} +)}; \frac{Mi\left\{ GFP^{E\text{.}3xP3}=ET1 \right\}CG{32017}^{MB02477}}{(Mi\left\{ GFP^{E\text{.}3xP3}=ET1 \right\}CG{32017}^{MB02477}\text{or} +)}$ single ♀

x $\frac{y^{1}w^{67c23}}{Y}; \frac{P\left\{ y^{+t7.7}v^{+t1.8}=nanos\text{-}Cas9.R \right\}attP2}{(P\left\{ y^{+t7.7}v^{+t1.8}=nanos\text{-}Cas9.R \right\}attP2 \text{or} \text{+)}}; \frac{Mi\left\{ GFP^{E\text{.}3xP3}=ET1 \right\}CG{32017}^{MB02477}}{(Mi\left\{ GFP^{E\text{.}3xP3}=ET1 \right\}CG{32017}^{MB02477}\text{or} \text{+)}}$ single ♂

Established multiple lines; retained a line producing no y^–^ or non-GFP progeny for use as the stock in the following cross

G3: $\frac{y^{1}w^{67c23}}{y^{1}w^{67c23}}; \frac{P\left\{ y^{+t7.7}v^{+t1.8}=nanos\text{-}Cas9.R \right\}attP2}{P\left\{ y^{+t7.7}v^{+t1.8}=nanos\text{-}Cas9.R \right\}attP2}; \frac{Mi\left\{ GFP^{E\text{.}3xP3}=ET1 \right\}CG{32017}^{MB02477}}{Mi\left\{ GFP^{E\text{.}3xP3}=ET1 \right\}CG{32017}^{MB02477}}$ ♀♀

x $\frac{y^{1}w^{*}}{Y}; \frac{TI\left\{ TI \right\}FRT101F iso}{TI\left\{ TI \right\}FRT101F iso}$ ♂♂

G4: $\frac{y^{1}w^{67c23}}{y^{1}w^{*}}; \frac{P\left\{ y^{+t7.7}v^{+t1.8}=nanos\text{-}Cas9.R \right\}attP2}{\text{+}}; \frac{Mi\left\{ GFP^{E\text{.}3xP3}=ET1 \right\}CG{32017}^{MB02477}}{TI\left\{ TI \right\}FRT101F iso}$ ♀♀

x $\frac{y^{1}w^{67c23}}{Y}; \frac{P\left\{ y^{+t7.7}v^{+t1.8}=nanos\text{-}Cas9.R \right\}attP2}{\text{+}}; \frac{Mi\left\{ GFP^{E\text{.}3xP3}=ET1 \right\}CG{32017}^{MB02477}}{TI\left\{ TI \right\}FRT101F iso}$ ♂♂

G5: $\frac{y^{1}w^{*}}{y^{1}w^{*}}; \frac{P\left\{ y^{+t7.7}v^{+t1.8}=nanos\text{-}Cas9.R \right\}attP2}{P\left\{ y^{+t7.7}v^{+t1.8}=nanos\text{-}Cas9.R \right\}attP2}; \frac{TI\left\{ TI \right\}FRT101F iso}{TI\left\{ TI \right\}FRT101F iso}$ ♀♀

x $\frac{y^{1}w^{*}}{Y}; \frac{P\left\{ y^{+t7.7}v^{+t1.8}=nanos\text{-}Cas9.R \right\}attP2}{P\left\{ y^{+t7.7}v^{+t1.8}=nanos\text{-}Cas9.R \right\}attP2}; \frac{TI\left\{ TI \right\}FRT101F iso}{TI\left\{ TI \right\}FRT101F iso}$ ♂♂

Selected stock for absence of y^–^ progeny

The following genetic scheme shows how we generated the stock used in G0 of the screen with a miniwhite-marked transgene insertion on the third chromosome (chosen for the visible marker and the vigor of the stock) and a dominant fourth chromosome marker on the fourth chromosome

G0: 9549 $\frac{w^{1118}}{w^{1118}}; \frac{P\left\{ w^{+mC}=ActGFP \right\}unc\text{-}{13}^{GJ}}{In\left( 4 \right)ci^{D}\text{,} ci^{D}pan^{ciD}}$ ♀♀ x 24640 $\frac{w^{1118}}{Dp\left( 2:Y \right)G\text{,} P\left\{ w^{+mC}=hs\text{-}hid \right\}Y}; \frac{MKRS}{TM2\text{,} y^{+}}$ ♂♂

G1: 24932 $\frac{y^{1}w^{*}}{y^{1}w^{*}}; \frac{P\left\{ w^{+mC}=EP \right\}G16942}{P\left\{ w^{+mC}=EP \right\}G16942}$ ♀♀ x $\frac{w^{1118}}{Dp\left( 2:Y \right)G\text{,} P\left\{ w^{+mC}=hs\text{-}hid \right\}Y}; \frac{\text{+}}{MKRS}; \frac{In\left( 4 \right)ci^{D}\text{,} ci^{D}pan^{ciD}}{\text{+}}$ ♂♂

G2: $\frac{y^{1}w^{*}}{w^{1118}}; \frac{P\left\{ w^{+mC}=EP \right\}G16942}{MKRS}; \frac{\text{+}}{In\left( 4 \right)ci^{D}\text{,} ci^{D}pan^{ciD}}$ ♀♀

x $\frac{y^{1}w^{*}}{Dp\left( 2:Y \right)G\text{,} P\left\{ w^{+mC}=hs\text{-}hid \right\}Y}; \frac{P\left\{ w^{+mC}=EP \right\}G16942}{MKRS}; \frac{\text{+}}{In\left( 4 \right)ci^{D}\text{,} ci^{D}pan^{ciD}}$ ♂♂

G3: $\frac{y^{1}w^{*}}{y^{1}w^{*}}; \frac{P\left\{ w^{+mC}=EP \right\}G16942}{P\left\{ w^{+mC}=EP \right\}G16942}; \frac{\text{+}}{In\left( 4 \right)ci^{D}\text{,} ci^{D}pan^{ciD}}$ ♀♀

x $\frac{y^{1}w^{*}}{Dp\left( 2:Y \right)G\text{,} P\left\{ w^{+mC}=hs\text{-}hid \right\}Y}; \frac{P\left\{ w^{+mC}=EP \right\}G16942}{P\left\{ w^{+mC}=EP \right\}G16942}; \frac{\text{+}}{In\left( 4 \right)ci^{D}\text{,} ci^{D}pan^{ciD}}$ ♂♂

Maintained by selecting for *ci^D^*

The following genetic scheme shows how we generated the stock used in G2 with a homozygous viable and fertile, miniwhite-marked transgene insertion on the second chromosome (chosen for the visible marker and the vigor of the stock) and a dominant fourth chromosome marker on the fourth chromosome.

G0: 24488 $\frac{y^{1}M\left\{ RFP^{3xP3\text{.}PB}GFP^{E\text{.}3xP3}=vas\text{-}int.Dm \right\}ZH\text{-}2A w^{*}}{y^{1}M\left\{ RFP^{3xP3\text{.}PB}GFP^{E\text{.}3xP3}=vas\text{-}int.Dm \right\}ZH\text{-}2A w^{*}}; \frac{M\left\{ 3xP3\text{-}RFP.attP \right\}ZH\text{-}102D}{M\left\{ 3xP3\text{-}RFP.attP \right\}ZH\text{-}102D}$ ♀♀

x 24639 $\frac{w^{1118}}{Dp\left( 2:Y \right)G\text{,} P\left\{ w^{+mC}=hs\text{-}hid \right\}Y}; \frac{wg^{Sp\text{-}1}}{CyO}$ ♂♂

G1: 26964 $\frac{y^{1}w^{*}}{y^{1}w^{*}}; \frac{P\left\{ w^{+mC}=EP \right\}MESR3^{G19459}}{P\left\{ w^{+mC}=EP \right\}MESR3^{G19459}}$ ♀♀

x $\frac{y^{1}M\left\{ RFP^{3xP3\text{.}PB}GFP^{E\text{.}3xP3}=vas\text{-}int.Dm \right\}ZH\text{-}2A w^{*}}{Dp\left( 2:Y \right)G\text{,} P\left\{ w^{+mC}=hs\text{-}hid \right\}Y}; \frac{\text{+}}{CyO}; \frac{M\left\{ 3xP3\text{-}RFP.attP \right\}ZH\text{-}102D}{\text{+}}$ ♂♂

G2: 26964 $\frac{y^{1}w^{*}}{y^{1}w^{*}}; \frac{P\left\{ w^{+mC}=EP \right\}MESR3^{G19459}}{P\left\{ w^{+mC}=EP \right\}MESR3^{G19459}}$ ♀♀

x $\frac{y^{1}w^{*}}{Dp\left( 2:Y \right)G\text{,} P\left\{ w^{+mC}=hs\text{-}hid \right\}Y}; \frac{P\left\{ w^{+mC}=EP \right\}MESR3^{G19459}}{CyO}; \frac{M\left\{ 3xP3\text{-}RFP.attP \right\}ZH\text{-}102D}{\text{+}}$ ♂♂

G3: $\frac{y^{1}w^{*}}{y^{1}w^{*}}; \frac{P\left\{ w^{+mC}=EP \right\}MESR3^{G19459}}{P\left\{ w^{+mC}=EP \right\}MESR3^{G19459}}; \frac{M\left\{ 3xP3\text{-}RFP\text{.}attP \right\}ZH\text{-}102D}{\text{+}}$ ♀♀

x $\frac{y^{1}w^{*}}{Dp\left( 2:Y \right)G\text{,} P\left\{ w^{+mC}=hs\text{-}hid \right\}Y}; \frac{P\left\{ w^{+mC}=EP \right\}MESR3^{G19459}}{P\left\{ w^{+mC}=EP \right\}MESR3^{G19459}}; \frac{M\left\{ 3xP3\text{-}RFP.attP \right\}ZH\text{-}102D}{\text{+}}$ ♂♂

G4: $\frac{y^{1}w^{*}}{y^{1}w^{*}}; \frac{P\left\{ w^{+mC}=EP \right\}MESR3^{G19459}}{P\left\{ w^{+mC}=EP \right\}MESR3^{G19459}}; \frac{M\left\{ 3xP3\text{-}RFP.attP \right\}ZH\text{-}102D}{M\left\{ 3xP3\text{-}RFP\text{.}attP \right\}ZH\text{-}102D}$ ♀♀

x $\frac{y^{1}w^{*}}{Dp\left( 2:Y \right)G\text{,} P\left\{ w^{+mC}=hs\text{-}hid \right\}Y}; \frac{P\left\{ w^{+mC}=EP \right\}MESR3^{G19459}}{P\left\{ w^{+mC}=EP \right\}MESR3^{G19459}}; \frac{M\left\{ 3xP3\text{-}RFP.attP \right\}ZH\text{-}102D}{M\left\{ 3xP3\text{-}RFP.attP \right\}ZH\text{-}102D}$ ♂♂

Selected stock for absence of non-RFP progeny

**Alternative approach for CRISPR mutagenesis**

The following genetic scheme shows how we generated mutations on an FRT-bearing fourth chromosome in the female germ line using guide RNA constructs located on the third chromosome and established the new mutations in stable stocks. Again, the guide RNA constructs (Supplementary Table 1) are shown generically as *P{U6-gRNA}.*

G0: $\frac{y^{1}w^{*}}{y^{1}w^{*}}; \frac{Dr^{1}}{TM6C\text{,} Sb^{1}}; \frac{TI\left\{ GMR\text{-}HMS04515 \right\}Gat^{eya}}{In\left( 4 \right)ci^{D}\text{,} ci^{D}pan^{ciD}}$ ♀♀ x $\frac{y^{2}cho^{1}v^{1}}{Y}; \frac{P\left\{ y^{+t7.7}v^{+t1.8}=U6\text{-}gRNA \right\}attP2}{P\left\{ y^{+t7.7}v^{+t1.8}=U6\text{-}gRNA \right\}attP2}$ ♂♂

G1: $\frac{y^{1}w^{*}}{y^{1}w^{*}}; \frac{P\left\{ y^{+t7.7}v^{+t1.8}=nanos\text{-}Cas9\text{.}R \right\}attP40}{CyO}; \frac{TI\left\{ TI \right\}FRT101F}{TI\left\{ TI \right\}FRT101F}$ ♀♀

x $\frac{y^{1}w^{*}}{Y}; \frac{TM6C\text{,} Sb^{1}}{P\left\{ y^{+t7.7}v^{+t1.8}=U6\text{-}gRNA \right\}attP2}; \frac{In\left( 4 \right)ci^{D}\text{,} ci^{D}pan^{ciD}}{\text{+}}$ ♂♂

G2: $\frac{y^{1}w^{*}}{y^{1}w^{*}}; \frac{P\left\{ y^{+t7.7}v^{+t1.8}=nanos\text{-}Cas9.R \right\}attP40}{\text{+}}; \frac{\text{+}}{P\left\{ y^{+t7.7}v^{+t1.8}=U6\text{-}gRNA \right\}attP2}; \frac{TI\left\{ TI \right\}FRT101F}{In\left( 4 \right)ci^{D}\text{,} ci^{D}pan^{ciD}}$ ♀♀

x 90852 $\frac{y^{1}w^{*}}{Y}; \frac{TI\left\{ GMR\text{-}HMS04515 \right\}Gat^{eya}}{In\left( 4 \right)ci^{D}\text{,} ci^{D}pan^{ciD}}$ ♂♂

G3: 90852 $\frac{y^{1}w^{*}}{y^{1}w^{*}}; \frac{TI\left\{ GMR\text{-}HMS04515 \right\}Gat^{eya}}{In\left( 4 \right)ci^{D}\text{,} ci^{D}pan^{ciD}}$ ♀♀ x $\frac{y^{1}w^{*}}{Y}; \frac{TI\left\{ TI \right\}FRT101F mutation}{TI\left\{ GMR\text{-}HMS04515 \right\}Gat^{eya}}$ single ♂

G4: $\frac{y^{1}w^{*}}{y^{1}w^{*}}; \frac{In\left( 4 \right)ci^{D}\text{,} ci^{D}pan^{ciD}}{TI\left\{ TI \right\}FRT101F mutation}$ ♀♀ x $\frac{y^{1}w^{*}}{Y}; \frac{In\left( 4 \right)ci^{D}\text{, }ci^{D}pan^{ciD}}{TI\left\{ TI \right\}FRT101F mutation}$ ♂♂

Mutation-bearing fourth chromosomes were established in homozygous stocks when possible.

The screen involving a guide RNA construct on the second chromosome was similar.

G0: $\frac{y^{1}w^{*}}{y^{1}w^{*}}; \frac{wg^{Sp\text{-}1}}{SM6a}; \frac{TI\left\{ GMR\text{-}HMS04515 \right\}Gat^{eya}}{In\left( 4 \right)ci^{D}\text{,} ci^{D}pan^{ciD}}$ ♀♀ x $\frac{y^{2}cho^{1}v^{1}}{Y}; \frac{P\left\{ y^{+t7.7}v^{+t1.8}=U6\text{-}gRNA \right\}attP40}{P\left\{ y^{+t7.7}v^{+t1.8}=U6\text{-}gRNA \right\}attP40}$ ♂♂

G1: $\frac{y^{1}w^{*}}{y^{1}w^{*}}; \frac{P\left\{ y^{+t7.7}v^{+t1.8}=nanos\text{-}Cas9.R \right\}attP2}{TM6C\text{,} Sb^{1}}; \frac{TI\left\{ TI \right\}FRT101F}{TI\left\{ TI \right\}FRT101F}$ ♀♀

x $\frac{y^{1}w^{*}}{Y}; \frac{SM6a}{P\left\{ y^{+t7.7}v^{+t1.8}=U6\text{-}gRNA \right\}attP40}; \frac{In\left( 4 \right)ci^{D}\text{,} ci^{D}pan^{ciD}}{\text{+}}$ ♂♂

G2: $\frac{y^{1}w^{*}}{y^{1}w^{*}}; \frac{\text{+}}{P\left\{ y^{+t7.7}v^{+t1.8}=U6\text{-}gRNA \right\}attP40}; \frac{P\left\{ y^{+t7.7}v^{+t1.8}=nanos\text{-}Cas9.R \right\}attP2}{\text{+}}; \frac{TI\left\{ TI \right\}FRT101F}{In\left( 4 \right)ci^{D}\text{,} ci^{D}pan^{ciD}}$ ♀♀

x 90852 $\frac{y^{1}w^{*}}{Y}; \frac{TI\left\{ GMR\text{-}HMS04515 \right\}Gat^{eya}}{In\left( 4 \right)ci^{D}\text{,} ci^{D}pan^{ciD}}$ ♂♂

G3: 90852 $\frac{y^{1}w^{*}}{y^{1}w^{*}}; \frac{TI\left\{ GMR\text{-}HMS04515 \right\}Gat^{eya}}{In\left( 4 \right)ci^{D}\text{,} ci^{D}pan^{ciD}}$ ♀♀ x $\frac{y^{1}w^{*}}{Y}; \frac{TI\left\{ TI \right\}FRT101F mutation}{TI\left\{ GMR\text{-}HMS04515 \right\}Gat^{eya}}$ single ♂

G4: $\frac{y^{1}w^{*}}{y^{1}w^{*}}; \frac{In\left( 4 \right)ci^{D}\text{,} ci^{D}pan^{ciD}}{TI\left\{ TI \right\}FRT101F mutation}$ ♀♀ x $\frac{y^{1}w^{*}}{y^{1}w^{*}}; \frac{In\left( 4 \right)ci^{D}\text{,} ci^{D}pan^{ciD}}{TI\left\{ TI \right\}FRT101F mutation}$ ♂♂

Again, mutation-bearing fourth chromosomes were established in homozygous stocks when possible.

In all these screens, the FRT-bearing fourth chromosome was derived from a stock related to the stock used in the crosses in the first section, but no effort was made to isolate a single fourth chromosome from the progenitor stocks for these crosses. Likewise, the X, Y, second and third chromosomes in the final stocks have mixed origins. The stable stocks providing females for the G0 and G1 crosses were generated ahead of time by standard crosses with readily available stocks and will not be detailed here.

**Molecular characterization of mutations**

We extracted genomic DNA from five flies from each stock potentially carrying a new mutation by crushing the flies in 75 μl of a buffered solution containing Proteinase K (10 mM Tris-Cl pH 8.0, 25 mM NaCl, 1 mM EDTA, 0.2 μg/μl Proteinase K), incubating the mixture at 37° for 30 minutes and then heat-killing the enzyme by incubating the mixture for 5 minutes at 95°. The supernatant containing genomic DNA was used for all subsequent steps. DNA concentrations were determined using a Thermo Scientific NanoDrop One spectrophotometer. For PCR amplification, the genomic preps were diluted in 10 mM Tris-Cl pH 8.0 to a DNA concentration of 150 ng/μl. We used LongAmp Taq 2X Master Mix from New England Biolabs (NEB) and reaction conditions recommended by the NEB Tm Calculator tool (https://tmcalculator.neb.com/). We confirmed the presence of the FRT-bearing transgene on the fourth chromosome by PCR amplification of the DNA preparation using a genomic primer (TTCGGTAAGGGGGCATACATATTT) and a primer within the FRT sequence (GGACAACACGGAAGTTCCTATTCTC) followed by electrophoresis in a 1% agarose gel. To determine if a mutation had been induced by CRISPR mutagenesis, we used primers flanking the target sites to produce ~600 base-pair products. PCR products were cleaned up using the Thermo Scientific GeneJET PCR Purification Kit (Product #K0701) and quantified using the NanoDrop One spectrophotometer. The purified PCR products were diluted to 30 ng/μl in 10 mM Tris-Cl pH8.0 in preparation for Sanger sequencing by Eurofins Genomics using the SimpleSeq Premixed Kit. We typically used the same primers for sequencing that we used to amplify the PCR products, but Supplementary Table 2 shows instances in which we used alternative primers for this step. Sequence from the mutated fourth chromosomes was aligned to the reference genomic sequence surrounding the gRNA target site using SnapGene software (Dotmatics). We could determine the sequences of mutations in DNA samples from homozygotes with no further steps. In DNA samples from heterozygotes, the presence of a mutation was detected by the divergence of sequence traces from the wild type and mutation-bearing chromosomes near the site of the mutation, leading to double-peak calls. The sequences of the normal and mutation-bearing chromosomes could often be extracted with the assistance of Poly Peak Parser (Hill *et al.* 2014), software that computationally separates mixed sequence traces in relation to a reference sequence. We retained stocks with mutations for further analysis.

**Characterization of lethal, female sterile and morphological phenotypes**

To provide a preliminary phenotypic characterization of the new fourth chromosome mutations, we assessed the viability of homozygous adults—generally with any survival judged as “viable”. In most cases, we did not attempt to measure relative viability. Viable adults were examined for grossly abnormal morphological defects (rough eyes, disturbed bristles or veins, abnormal coloration, etc.) and females were tested for fertility with the production of any progeny judged as “fertile”, i.e. we did not measure relative fecundity. We assessed male fertility for only a few mutations.

We also assessed viability, morphological phenotypes and female fertility of adults with the new fourth chromosome mutations combined with preexisting mutations or chromosomal deletions for the regions of the targeted genes using the same broad criteria (summarized in Supplementary Table 9 with cross details provided in Supplementary Table 8). Again, we assessed male fertility for only a few genotypes.

For several genes, the phenotypes associated with preexisting mutations had never been described in publications. When a phenotypic analysis of such mutations was judged informative, we assessed viability, morphological phenotypes and female fertility of adults with these mutations combined with chromosomal deletions for the regions of the mutations. These results are reported in Supplementary Table 7 and mentioned, when relevant, in Supplementary Table 10.

**References cited in this supplementary file**

Cook, R. K., M. E. Deal, J. A. Deal, R. D. Garton, C. A. Brown *et al.*, 2010 A new resource for characterizing *X*-linked genes in *Drosophila melanogaster*: systematic coverage and subdivision of the *X* chromosome with nested, *Y*-linked duplications. Genetics 186**:** 1095-1109.

Hill, J. T., B. L. Demarest, B. W. Bisgrove, Y. C. Su, M. Smith *et al.*, 2014 Poly peak parser: Method and software for identification of unknown indels using sanger sequencing of polymerase chain reaction products. Dev Dyn 243**:** 1632-1636.

Ryder, E., F. Blows, M. Ashburner, R. Bautista-Llacer, D. Coulson *et al.*, 2004 The DrosDel collection: a set of P-element insertions for generating custom chromosomal aberrations in *Drosophila melanogaster*. Genetics 167**:** 797-813.
